# Women in Saudi Arabia and the Prevalence of Cardiovascular Risk Factors: A Systematic Review

**DOI:** 10.1155/2016/7479357

**Published:** 2016-09-29

**Authors:** Mashael K. Alshaikh, Filippos T. Filippidis, Juren P. Baldove, Azeem Majeed, Salman Rawaf

**Affiliations:** ^1^Department of Primary Care and Public Health, School of Public Health, Faculty of Medicine, Imperial College London, London, UK; ^2^Department of Pharmacy, King Saud University Medical City, Riyadh, Saudi Arabia; ^3^Department of Critical Care, King Saud University Medical City, Riyadh, Saudi Arabia

## Abstract

*Background*. Cardiovascular disease (CVD) is one of the leading causes of death in Saudi Arabia. Saudi women in particular are more susceptible as there are sociocultural restrictions on female physical activities that may lead to high prevalence of CVD risks, especially obesity, and physical inactivity. This study aims to systematically review the published articles related to the prevalence of CVD risk among women in Saudi Arabia. The search strategy covers all published articles that assess the risk factor of CVD in Saudi Arabia from January 2000 to December 2015, using the following sources: Medline, Embase, and PsycINFO. A total of 61 studies were included.* Results.* Prevalence among Saudi women of smoking ranged from 1.1% to 9.1%, hypertension was 21.8%, diabetes ranged from 9.6% to 27.6%, overweight was 27%, and obesity was 40.23%, and physical inactivity ranged from 53.2% to 98.1%. Hypercholesterolemia prevalence on Saudi women on average was 24.5%, while metabolic syndrome ranged from 13.6% to 40.3%.* Conclusion*. The prevalence of CVD risk factors is high among women in Saudi Arabia especially in obesity and physical inactivity. Public health authorities must implement solutions from a gender specific aspect to reverse the trend and decrease the prevalence of CVDs among Saudi women.

## 1. Introduction 

Cardiovascular disease (CVD) is a major public health problem, and one of the main causes of death globally [1]. According to the World Health Organization (WHO) in 2011, CVD accounted for 17.3 million deaths per year, and by 2030 this number is expected to grow to more than 23.6 million [1, 2].

There are a number of established measurable risk factors, for example, diabetes, hypertension, and obesity that may lead to the development of CVD events. These risk factors are aspects of a patient's style of living that can be modified. Knowing the risk factors is a useful approach for identifying people at high risk who will most benefit from counselling and clinical management of their risk factors [3]. In recent years, age-adjusted CVD mortality has been cut in half in developed countries due to a decrease in associated risk factors [4]. Therefore, focusing on reducing risk factors is a major improvement in terms of developing strategies for preventing CVD [5], and this can explain the decline in CVD mortality and morbidity [6].

The WHO has developed specific strategies to have a paradigm shift from CVD treatment to CVD prevention; this approach is recommended for CVD prevention worldwide [5]. Effective, integrated health promotion policies and programmes are one of the best tools to counteract the epidemic of obesity, diabetes, and other CVD risks that are emerging worldwide [7]. Applying these recommendations helps to shift the focus towards the prevention of primary risk factors, and in the long term it can lead to health improvement and decrease CVD [4].

However, in the Arab Gulf region, around 50% of the deaths of those aged below 70 were found to be attributed to CVD deaths, while in Western countries (UK, Germany and the US), it is about 25%, a dramatic difference [8]. This is particularly true for CVD deaths from ischemic heart disease, obesity, and diabetes complications [9, 10]. In 2008, there were more than one million deaths in Arab countries from noncommunicable diseases, which accounted for 60% of all deaths [11]. Aljefree and Ahmed conducted a systematic review on the prevalence of CVD and its associated risk factors among the adult population in the Gulf region, and they concluded that there was a high prevalence of CVD risks in Gulf countries, particularly regarding obesity among women in this region [12].

Saudi Arabia is the largest country on the Arabian Peninsula, extending over an area of 2,150,000 km^2^, with a population of more than 30 million [13]. It is one of the rapidly developing countries, adapting an increasingly urbanised lifestyle [14]. The country has reported an increase in CVDs parallel to that of other rapidly developing economies. The most predominant CVD risk factors include diabetes mellitus, obesity, hypertension, sedentary lifestyle, and smoking. The increasing prevalence of these risk factors has led to a growing incidence of ischaemic heart disease and heart failure [15]. The Saudi Project for Assessment of Coronary Events (SPACE) Registry, the first registry in Saudi Arabia that investigates patients with acute coronary syndrome (ACS), found that more than half of the patients admitted with ACS had diabetes, and around half were having hypertension. They also found that patients diagnosed with ACS in Saudi Arabia are 8 to 11 years younger than the homologous patients in ACS registries found in developed countries [16].

Women in Saudi Arabia are not allowed to drive, and they are required to have a guardian for transportation purposes [17]. Furthermore, there is no sports education in girls' schools and it is prohibited by social norms for females to practice physical activities in public schools [18]. All of these sociocultural factors have created unhealthy lifestyles, which have become part of the social norms within Saudi society, thereby increasing the prevalence of sedentary lifestyle and obesity in women living in Saudi Arabia [19].

This paper aims to review the published works related to the prevalence of CVD risk among women in Saudi Arabia. Cardiovascular disease affects many women in Saudi Arabia, and for this reason, it is important to decrease the burden of the risk and to identify the most dominant factors among women in Saudi Arabia. Until now, there has been no systematic review, studying the prevalence of CVD risk among adult women in different regions and populations, for example, among university students, those attending primary care clinics and from the national studies in Saudi Arabia.

## 2. Methods

### 2.1. Review Question

A literature exploration was used to identify relevant published studies of prevalence of CVD risk factors, in order to answer the following question: what is the prevalence of obesity, diabetes, hypertension, hypercholesterolemia, physical inactivity, and metabolic syndrome among women living in Saudi Arabia?

### 2.2. Search Strategy

The search strategy intended to cover all published literature written in English and Arabic that look at the risk factors of CVD in Saudi Arabia. The target articles covered the period from January 2000 to December 2015. This period was chosen in order to account for advances both in treatments and in medical technology.

A three-step search strategy was utilized in this review using the following sources: Medline (January 2000 to December 2015); Embase (January 2000 to December 2015); PsycINFO (January 2000 to December 2015). Step 1: the search was undertaken across all the databases using all of the identified keywords. Step 2: the titles and abstracts of all the articles of potential interest were reviewed for the inclusion and exclusion of studies. Step 3: the reference lists of all identified reports and articles were searched for additional relevant studies. We used combinations of medical subject headings (MESH) using the following search terms (see Box 1).

### 2.3. Selection of Studies

After applying the aforementioned three steps of the search strategy, two reviewers (Mashael K. Alshaikh and Juren P. Baldove) independently screened the titles and abstracts of the initially identified studies to determine whether they would satisfy the selection criteria. Full-text articles were retrieved for the selected titles. The reference lists of the retrieved articles were searched for additional publications.

All studies, wherein body mass index (BMI), obesity, metabolic syndrome, hypertension, dyslipidaemia, and physical inactivity were investigated, were eligible for inclusion. No limitations on publication type, status, or study design were imposed. However, we did not include secondary reports such as review articles without novel data synthesis. The inclusion criteria required that the studies had been carried out in Saudi Arabia, and included populations comprised of adults aged above 15 years old; both resident and expatriate populations and urban and rural populations were included. Studies of the general population, those working, students, those attending healthcare, and other populations were included. We did not specify diagnostic criteria for the studied conditions but incorporated them into our data synthesis.

### 2.4. Data Extraction/Quality Assessment

A data collection form was designed prior to the implementation of the search strategy. Two reviewers extracted independently the relevant information from the selected studies (Mashael K. Alshaikh and Juren P. Baldove). The data collection form included author name and published year, study location, population, repose rate, region, study type, sample size, and sampling method. The tools used are illustrated in Table 1.

We excluded all review articles, studies on children and adolescents, studies not undertaken in Saudi Arabia, and duplicates. The Newcastle-Ottawa Scale (NOS) was used to assess the quality of nonrandomised studies [80]. This NOS awards a maximum of nine stars to each study: four stars for the adequate selection of cohort studies, two stars for comparability of cohort studies on the basis of the design and analysis, and three stars for the assessing the outcome. We defined studies with NOS of ≥6 stars as moderate- to high-quality studies and studies with a NOS of <6 stars as low-quality studies. Criteria for quality assessment and characteristics for each single study are shown in Table 2 (see Supplementary file^*∗*^ in Supplementary Material available online at http://dx.doi.org/10.1155/2016/7479357).

## 3. Results

**Figure 1:**
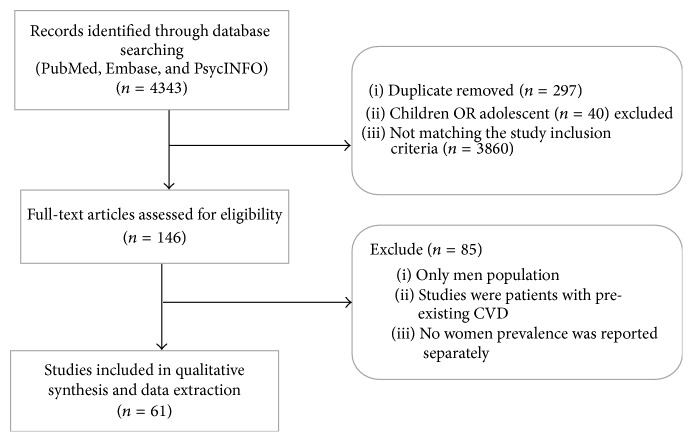
Flow chart of study selection.

Sixty-one studies were included in the systematic review that investigated the prevalence of CVD risk among women in Saudi Arabia (Figure 1: flow chart of study selection).

### 3.1. Cigarette Smoking

Seventeen studies reported on the prevalence of smoking among women. Ten studies were undertaken among university students [20, 23, 25, 26, 29, 30, 32–35], two among employees [37, 38], one at a leisure place [40], one among people presenting in hospital [47], and three studies within national or regional populations [66, 67, 79]. The prevalence of smoking ranged from 1.1% to 9.1% in the above populations.

### 3.2. Hypertension (BP ≥ 140/90 mmHg)

**Figure 2:**
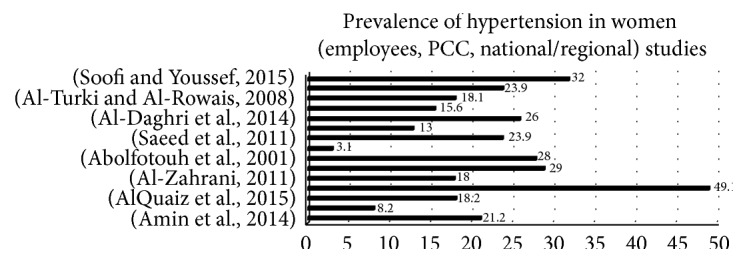
Prevalence of hypertension in women (employees, primary care centres, and national studies).

The prevalence of hypertension was reported in seventeen studies. Three were among university students [22, 25, 28], two were among employees [36, 37], two others were among healthy volunteers [40, 41], four were from primary care centers (PCCs) and health clinics [47, 51–53], and seven were conducted on a national or regional level [14, 57, 59, 62, 69, 70, 75]. Hypertension prevalence among university students was only 2.7%, while the average prevalence among women was 21.8%, the majority of whom were over age 40, from the PCCs and national studies (see Figure 2).

### 3.3. Diabetes

**Figure 3:**
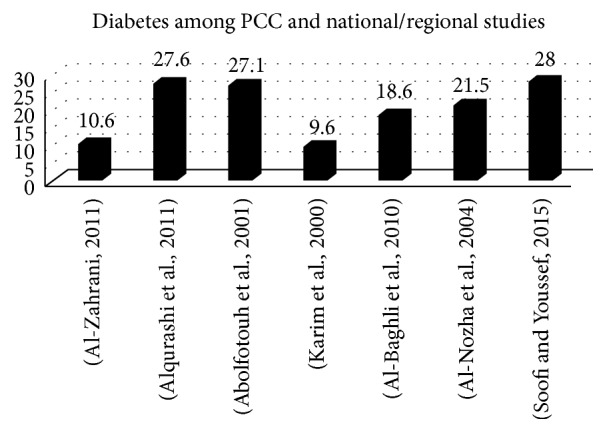
Prevalence of hypertension in women (employees, primary care centres, and national studies).

Twelve studies reported the prevalence of diabetes. Diabetes is an age-prevalent disease; as men or women get older, this increases the chance of having this disease detected. Two studies that were conducted among university students showed low prevalence, ranging from 1% to 2.1% [22, 25], while among employees and healthy volunteers the diabetes prevalence ranged from 4% to 5.2% [36, 37, 40]. Seven studies reported on the prevalence among patients from the PCCs and at a national level that ranged from 9.6% to 27.6%, most of them being over age 40 [14, 47, 48, 52, 54, 63, 78] (see Figure 3).

### 3.4. Overweight and Obesity

**Figure 4:**
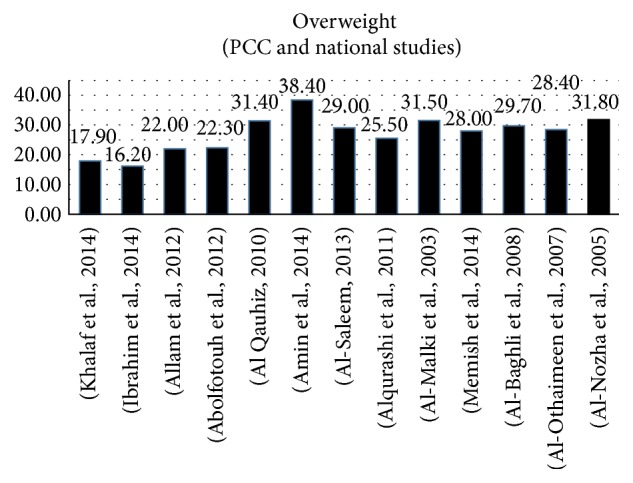
Prevalence of overweight in women (primary care centres and national studies).

Thirteen studies reported the rate of being overweight in women. Five studies were carried out among university students [21, 22, 27, 28, 31], one was among employees [36], three were in PCCs [46, 48, 50], and four were done on a national or regional level [58, 72, 73, 76]. The average prevalence of overweight among women was 27%, and it ranged from 16.2% to 38.4% (see Figure 4).

**Figure 5:**
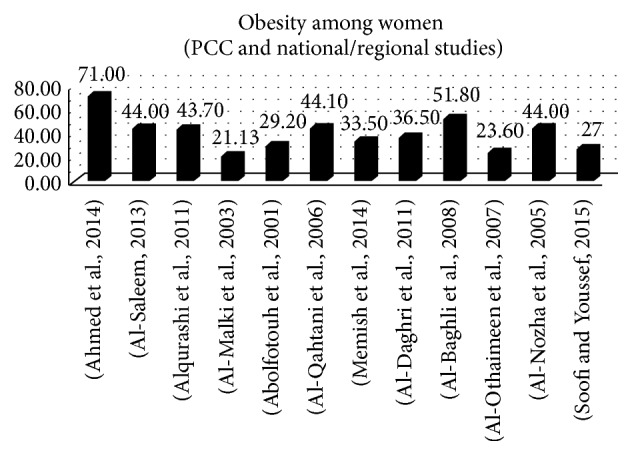
Prevalence of obesity in women (primary care centres and national studies).

Twenty studies reported the prevalence of obesity, six among university students [21, 22, 25, 27, 28, 31], and the prevalence of obesity ranged from 5.7% to 29% with this population. Two studies were conducted with employees and healthy volunteers, where the prevalence of obesity was 57.1% and 46.7%, respectively. Seven studies were conducted in PCCs [45, 46, 48, 50, 52, 55, 56] and six on a national or regional level [14, 58, 65, 72, 73, 76]. The average prevalence within PCCs and national studies was 40.23%, and it ranged between 21.13 and 71% (see Figure 5).

### 3.5. Hypercholesterolemia

Nine studies reported the prevalence of hypercholesterolemia; two were among students [25, 28], three were among employees [36, 37, 39], and one was in PCCs [49]. Three further studies were carried out on a national level [60, 64, 71]. There were various cut-off points in defining hypercholesterolemia used among the studies, which reduces their comparability. For example, in the two national data studies, [64, 71] hypercholesterolemia was defined as levels of cholesterol >5.2 mmol/L, while Basulaiman et al. [60] used a measure of >6.2 mmol/L. The average of hypercholesterolemia prevalence in all the studies was found to be 24%.

### 3.6. Physical Inactivity

The prevalence of physical inactivity was reported in seven studies [14, 24, 25, 27, 37, 44, 74]. The rates ranged from 53.2% to 98.1%, and one study was done on a national level [74].

### 3.7. Metabolic Syndrome

Six studies reported on the prevalence of metabolic syndrome, four of which used the National Cholesterol Education Program Adult Treatment Panel (NCEP ATP) III definition [56, 62, 68, 77]. Metabolic syndrome rates ranged from 13.6% to 40.3%, reporting on a national level and from patients attending PCCs. Two studies used the International Diabetes Federation (IDF) definition [42, 61] in which the rates ranged between 25.5% and 55%.

## 4. Discussion

Our review of the studies identifies a consistently high prevalence of obesity, diabetes, hypertension, and physical inactivity among women in Saudi Arabia, especially obesity and physical inactivity. These conditions can be affected or prevented by changes in behaviours and lifestyles. We noted that almost half of the women population are obese and three quarter are not physically active. The majority of studies have been done in the central region which may be due to the higher population density in that area, followed by the eastern and western regions, while the least has been done in the northern region. The overall quality of the included studies varied, half of the studies were high- to moderate-quality score (6—9), and the rest were lower score (5—1), whereas five studies score ≤2.

### 4.1. Obesity

Obesity predisposes a person to a number of cardiovascular risk factors, including diabetes, hypertension, and dyslipidaemia. The overall prevalence of obesity in adult females in Saudi Arabia is one of the highest amongst females, worldwide [12]. The study used data from the Saudi national health survey showed that 28.7% of the population aged 15 years old and above were obese with prevalence of 24.1% among men and 33.5% among women. The risk of obesity in Saudi women, the study showed, increases with a number of risk factors including age, being married or previously married, being diagnosed with a chronic condition, and being prehypertensive or hypertensive. Moreover, women who were more educated are less likely to be obese than those who had a primary school educational level or less [58]. We found that the prevalence of being overweight varied between 16% and 36.4%, and for obesity 21.3% and 71%. This was more than the obesity levels reported in Lebanon and Tunisia, but less than in Kuwait and Egypt [81]. Moreover, the prevalence of overweight (26%) and obesity (29%) in Saudi women aged 20 to 39 years was higher than that in the USA [82].

### 4.2. Diabetes

Diabetes is well known to be a coronary artery disease risk equivalent, and also, many studies have demonstrated a worse outcome for diabetic patients with ACS compared with nondiabetic patients [83, 84]. According to the SPACE registry, more than half of the patients with ACS have diabetes [16]. Diabetes prevalence among the GCC countries is considered to be one of the highest in the world [85]. Besides, the prevalence of diabetes among Saudi Arabia's population increased by 15% over the period from 1987 to 2011 [86]. Data from the Saudi Health Interview Survey (SHIS) found that the prevalence of diabetes among Saudi women was at 11.7%. Although the recent findings showed that the prevalence of diabetes in Saudi women is decreasing, the demographic characteristics however like age and marital status were strongly associated with being borderline diabetic or diabetic. They are also more prone to be diabetic if they are older, separated, divorced, or widowed. The findings also showed that Saudi diabetic women do not receive a routine medical examination, self-rated as health-poor health, and a significant percentage of diabetic women at 48.4% were undiagnosed, and 27.8% of those receiving treatment had uncontrolled diabetes [87]. In this review, the average prevalence of diabetes among women was 20.4%. Saudi Arabia has one of the world's highest incident rates of diabetes; according to the International Diabetic Federation (IDF) there were 3.4 million diabetes cases within Saudi Arabia in 2015, and the prevalence of diabetes among adults aged 20–79 is 17.6% [88]. Furthermore, the incident rate of diabetes increases with the degree and duration of being overweight or obese as high prevalence of obesity and physical inactivity contribute to the development of insulin resistance and metabolic syndrome [89].

### 4.3. Hypertension

Aetiologically, coronary artery disease is closely tied to diabetes and hypertension. As mentioned previously according to SPACE registry more than 55% of the patients with ACS have hypertension [16]. In this review, the average prevalence of hypertension was 21.8%. The last SHIS report showed that prevalence of hypertension was 12.5% among Saudi women. Although the risk of being hypertensive was lower among females compared to men, however, it increases with age, being obese, diabetes, and hypercholesterolemia. Age and last routine medical exam were significantly associated with women having undiagnosed hypertension or having borderline hypertension or being hypertensive. The risk of being hypertensive was higher among women who were separated, divorced, or widowed. However, educated women with college degrees or higher are less likely to be hypertensive. Similarly, the findings on the prevalence of hypertension may be subsiding; still more than half of the hypertensive Saudis are undiagnosed at 57.8% and 55.0% of those on treatment were not controlled, respectively [59, 87]. Tailakh et al. reported the prevalence of hypertension among ten Arab countries including Saudi Arabia in their systematic review. The overall estimated prevalence was 29.5%. In five out of the thirteen studies women were more hypertensive than men. While two studies showed that men have a higher prevalence of hypertension, the rest of the studies reported no difference between genders [90]. Comparing to the USA, according to the US National Health and Nutrition Examination Survey, the prevalence of hypertension was 27.1% in men and 30.1% in women [91]. Although the prevalence of the hypertension amongst Arabs and Saudis is lower than in the US data, the number may be much more than what is reported according to Tailakh et al. review, and from the SHIS report, because the majority of the hypertensive patients were unaware of their hypertension, due to issues such as illiteracy [59, 90].

### 4.4. Physical Inactivity

Physical inactivity is globally more prevalent among women than among their male counterpart [92]. Similarly, physical inactivity and a sedentary lifestyle have been reported as high within Muslim countries, with an overall prevalence of 32.3%, and even higher in women 35.5% [93]. In addition, in GCC countries, Aljefree and Ahmed reported a low level of activity among women. In Saudi Arabia, women's activities are limited because of cultural and religious norms, and women are prohibited from driving and require a guardian for transportation to go to a place such as a health club. This increases the personal burden of attending and limits their physical activities. Further, there is no physical activity in the school curriculum for girls in Saudi public schools [17]. In our study, we found a high rate of reported physical inactivity, ranging between 53.2% and 98.1% more than the reported numbers for the opposite gender in the Saudi community [74].

### 4.5. Hypercholesterolemia

The prevalence of hypercholesterolemia among women in the GCC region ranged from 9% to 53.2% [12]. This review found that different studies used different measures of hypercholesterolemia. For example, Basulaiman et al. [60] defined hypercholesterolemia with 6.2 mmol/L as a cut-off point, while in Al-Nozha et al. [71] a measure of 5.2 mmol/L was used to define hypercholesterolemia. This could explain the higher prevalence in the Al-Nozha study [60, 71]. In addition, hypercholesterolemia is significantly associated along with age, type of fat consumed, obesity, and diagnosis history of hypertension and diabetes among Saudis [60]. Also, the prevalence of hypercholesterolemia among Saudi women was at 7.3% while women who watch five hours of television or more daily were twice as likely to be borderline hypercholesterolemic [87].

### 4.6. Smoking

The rate of cigarette smoking in women in the GCC region ranges from 0.5% to 20.7% [12]. This review revealed rates ranging from 2.5% to 9.1% in women, while among men the prevalence of smoking ranged from 11.6 to 52.3% [94]. The SHIS survey reported that approximately 1.4% of the population were daily smokers of both cigarette/cigar and shisha at 2.6% in men and 0.1% in women. Saudis aged 15 to 64 years who were smoking shisha increased from 3.34% to 7.35% in men and from 0.5 to 1.28% in women. The study also revealed that 23.3% of the Saudi population with 32.3% of men and 13.5% of women were exposed to second-hand smoke for at least one day during the prior 7 days at home, work, or school [79]. Although there are many reported surveys on the increasing number of female smokers, especially those smoking the hookah (water pipe), this review only covers the smoking of cigarettes. A systematic review carried out in 2011 reported that the prevalence of water pipe smoking in Saudi Arabia ranged from 9% to 10% [95]. The problem may even be greater, as many national surveys do not mention second-hand smoke among women or families as part of CVD risks nor is it culturally acceptable for women to smoke. The actual number of women smoking within Saudi Arabia could therefore be significantly underestimated.

### 4.7. Metabolic Syndrome

The metabolic syndrome is a cluster of risk factors (including obesity, dyslipidaemia, hypertension, and impaired glucose metabolism) that have been shown to be strongly predictive of cardiovascular disease [96]. Mabry et al. reported the prevalence of metabolic syndrome among men and women within the GCC countries ranged from 20.7% to 37.2% (ATPIII definition) and from 29.6% to 36.2% (IDF definition) for men and for women, from 32.1% to 42.7% (ATPIII definition) and from 36.1% to 45.9% (IDF definition) [97]. In this review, Saudi women had a prevalence close to women in other GCC countries with rates varying between 13.6% and 40.3% (ATPIII definition) and 25.5 and 55% (IDF definition).

### 4.8. Challenges Facing Saudi Women in Adapting Healthy Lifestyle

The majority of the reported studies from the national data shows that Saudis have high rates of diagnosed and undiagnosed chronic diseases, and both genders have similar concerns in terms of health seeking behaviour; about 22.4% only of Saudi women received a periodic health examination within the last 2 years as compared to 23.3% of men [98]. However, women in Saudi Arabia may have difficulty in engaging in a lifestyle as healthy (e.g., gym use) as they would like to be due to mobility restrictions. This is an important obstacle to deal with, as women in Saudi cannot drive and need the presence of a male relative to go to and avail the services of a healthcare facility. Though, over the past 10 years Saudi women have had growing participation in senior management positions and in the decision-making process in public and private sectors which may, over time, lead to improved health status for all women. Empowerment of women was the main focus of the Saudi government, under King Abdullah [99]. This emphasis could influence women's health and possibly give more power to women and change the social norm by forcing a focus on women's health needs and to facilitate the adaptation of healthier lifestyles.

## 5. Limitation

There was significant heterogeneity between studies with respect to definitions of risk factors, design, and population characteristics. In addition, the lack of standardization for the definition of dyslipidaemia limits our ability to provide summary estimates for this important risk factor. Likewise, the majority of the tools used for measuring physical inactivity varied between studies.

## 6. Conclusion 

This is the first systematic review to focus exclusively on the women's dimension of CVD risk factors and the unique social and cultural context in which CVD risk is evaluated and managed in Saudi women. There were several methodological challenges, in particular, the different populations studied and the methods used to assess the prevalence of CVD risks. In summary, the prevalence of CVD risk factors is high among women in Saudi Arabia, particularly obesity and physical inactivity. We need health promotion programs and reorientation of primary health care to improve CVD detection at earlier stages and improve its management. Public health authorities need to consider gender specific aspects of the problem in order to decrease the rising trend of CVD prevalence in Saudi Arabia implementing programs to influence change in social norms in order to create a healthier and more active society.

## Supplementary Material

Newcastle–Ottawa scale (NOS) is a tool used for assessing the quality of non-randomized studies included in a systematic review and/or meta-analyses. NOS awards a maximum of nine stars to each study: four stars for the adequate selection of cohort studies, two stars for comparability of cohort studies on the basis of the design and analysis, and three stars for the assessing the outcome.

## Figures and Tables

**Box 1:**
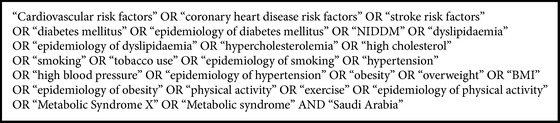
Selected search terms.

**Table 1:** Data extraction.

	Author/yrs.	Response rate/sampling method	Population	Outcome	Prevalence female%	Tools	*Q* score/9	Age group/sample size/% female	Location
1	Mahfouz et al., 2014 [20]	91.80%/the sample was stratified first according to the three institutions and then by colleges, and, finally, clusters of classes were randomly selected from each stratum	Students	Smoking	4.6%	WHO | Global youth tobacco survey (GYTS)	6	17–24/3764/35.7%	South

2	Khalaf et al., 2014 [21]	n/a/randomly selected female university students, multistage stratified random selection procedure	Students	Obesity	17.9% overweight 5.7% obese	Metabolic Equivalent of Task (MET)	7	Mean age of 20.4/663 female students (100% female)	South

3	Ibrahim et al., 2014 [22]	n/a/multistage stratified random sample method was used	Students	(1) HTN	(1) HTN = 5.8%	(i) JNC-7 (ii) WHO classification for DM	7	20–28 years/214 (75.2% females)	West
				(2) FBS	(2) High fasting level = 2.1%				
				(3) Obesity	(3) Overweight, 16.2 female Obese, 10.6 female				

4	AlSwuailem et al., 2014 [23]	67%/all register dental students in university	Students	Smoking	Female 2.4%	WHO-Global Adult Tobacco Survey (GATS) questionnaires	5	19–22 years old and above/400/42.5% female	Central

5	Khalaf et al., 2013 [24]	n/a/random multistage stratification	Students	Physically inactive	57% inactive. Only 43% of the participants met these guidelines	Arab Teens Life Style questionnaire (ATLS)	6	Mean age was 20.4 years/663/100%	South

6	Koura et al., 2012 [25]	97.6%/n/a	Students	(1) HTN	(1) 10/370 = 2.7%	(i) (WHOSTEPS) (ii) JNC7 criteria	6	<20 and >20/370/100%	East
				(2) DM	(2) 4/370 = 1.0%				
				(3) Smoking	(3) 5/370 = 1.35%				
				(4) Obesity	(4) Overweight/obesity: 29.1%				
				(5) High cholesterol	(5) 3.24% (tc > 200)				
				(6) Physically inactive	(6) 53.2% calculated				

7	Azhar and Alsayed, 2012 [26]	96.9%/randomly distributed among female students	Students	Smoking	4.2%	Global tobacco survey (GATS)	3	18–25/310/100%	West

8	Allam et al., 2012 [27]	97%/randomization through computer number	Students	(1) Obesity	(1) Overweight: *female 22/100 = 22*% Obese: *female 6/100 = 6*%	International physical activity questionnaire (IPAQ-SF)	5	18–26/394/50.7%	North/west
				(2) Physically inactive	(2) Physically inactive: *female 65/100 = 65*%				

9	Abolfotouh et al., 2012 [28]	n/a/n/a	Students	(1) Obesity	(1) Overweight: female 22.3% Obesity 30+: female 15.3%	n/a	3	18–26 years/501/23.55%	Central
				(2) HTN	(2) HTN = 13.6%				
				(3) High cholesterol	(3) High TCh female = 0.9%				

10	Wali, 2011 [29]	n/a/n/a	Students	Smoking	*9.1*%	Self-administered, Global Adult Tobacco Survey based questionnaire	5	<22–>24/643,411/(69%) females	West

11	Mandil et al., 2010 [30]	90%/sample was stratified according to college, and then clusters of classes	Students	Smoking	5.9%		5	17–25/6793/55.5%	Central

12	Al Qauhiz, 2010 [31]	99.8%/all university students	Students	Obesity	Overweight = 31.4% Obesity = 16.5	n/a	5	22–24/799/100%	Central

13	Subhan et al., 2009 [32]	84%/all students in medical science	Students	Smoking	12/305 female = 3.9%	n/a	4	18–37/941/69%	Central

14	Al-Turki and Al-Rowais, 2008 [33]	75.4%/all female students in medicine school	Students	Smoking	Current: 8/337 = 2.4%	n/a	1	n/a/337/100%	Central

15	Merdad et al., 2007 [34]	95.5%/all students	Students	Smoking	11%	Self-administered, Global Adult Tobacco Survey based questionnaire	5	18–26 years old/1050/100%	West

16	Hashim, 2000 [35]	91%/random	Students	Smoking	9%	n/a	2	18–26/647/40.8% (264)	Central

17	Amin et al., 2014 [36]	33.1%/all university's employees were eligible	Employees	(1) HTN	(1) 21.2%	World Health Organization STEPwise	7	24–63/691/28.7% (F (*N* = 198) Males (*N* = 493))	East
				(2) DM	(2) 4%				
				(3) Obesity	(3) Overweight 38.4%				
				(4) High cholesterol	(4) 20.2%				

18	Rehmani et al., 2013 [37]	71.6%/selected households were conducted from May to October 2010 at 2 National Guard housing complexes in the Eastern region	Employees	(1) HTN	(1) HTN = 8.2%	Health Measures Survey	6	14–34/2054/42.6%	East
				(2) DM	(2) DM = 5.2%				
				(3) Smoking	(3) Smoking female 2.1%				
				(4) Obesity + overweight	(4) Overweight + obesity = 57.1%				
				(5) High cholesterol	(5) High serum lipid = 17.7%				
				(6) Physical activity	(6) Practicing physical exercise 51.9%				

19	Siddiqui and Ogbeide, 2001 [38]	66%/all hospital staff	Employees	Smoking	Female = 8.3%		5	10–51/230/53%	Central

20	Abalkhail et al., 2000 [39]	76.6%/all university staff and a sample of school workers	Employees	High cholesterol	9.7%	NCEP	5	<35–>+40/1,649/(28.7%)	West

21	AlQuaiz et al., 2015 [40]	80%/convenience sampling strategy, Saudi women ≥ 30 years of age were invited to participate	Leisure places & healthy volunteers	(1) HTN	(1) HTN: 18.2%	Framingham Risk, Kaiser Physical Activity Survey (KPAS).	5	≥30/291/100%	Central
				(2) DM	(2) DM: 10%				
				(3) Smoking	(3) SMOK: 3.4%.				

22	Elkhalifa et al., 2011 [41]	n/a/randomly selected at a megamall	Leisure places & healthy volunteers	HTN	49.1%	n/a	3	<30–>50/243/53.9%	West

23	Al-Daghri et al., 2013 [42]	n/a/patients were recruited randomly from their homes using the cluster sampling	Leisure places & healthy volunteers	Mts	55%	Metabolic syndrome was determined according to the IDF	4	19–60/185/52.9%	Central

24	Habib, 2013 [43]	n/a/n/a	Leisure places & healthy volunteers	Obesity	Mean = 31.9 ± 10.7 = 46.7%	WHO classification BMI	1	18–72/530/31.5%	n/a

25	Amin et al., 2014 [44]	n/a/ten PHCs were randomly selected	PCC	Physical inactivity	58.5% inactive	Assessment of Intentional Leisure Time Physical Activity “ILTPA”: The Global Physical Activity Questionnaire (GPAQ)	7	18–78/2127/56% Women (*N* = 1193) Men (*N* = 934)	East

26	Ahmed et al., 2014 [45]	n/a/selected from 30/105 primary health care (PHC) centrals by simple random method	PCC	Obesity	71%	n/a	7	<25–+71/5000/50%	North

27	Al-Saleem et al., 2013 [46]	n/a/all PCCs in Aseer region	PCC	Obesity	Overweight female 29% Obese female 44%	WHO definition for BMI	5	18–65+/6917 female 3483/(50.4)%	South

28	Al-Zahrani, 2011 [47]	n/a/from patients presented for treatment at KAUFD-female section	PCC	(1) HTN	(1) 18%	JNC7	3	18–>50/208/100%	West
				(2) DM	(2) 10.6%				
				(3) Smoking	(3) 10/208 = 4.8%				

29	Alqurashi et al., 2011 [48]	n/a/all patients attending a primary care clinic	PCC	(1) DM	(1) 27.6% in females	IDF	3	12–19 years old, >70/6024/3714 (61.65%)	West
				(2) Obesity	(2) Overweight female = 25.5% Obese female = 43.7%				

30	Ogbeide et al., 2004 [49]	n/a/all patients above 13 years of age seen by the investigators in Al Kharj Health Centre	PCC	High cholesterol	43.3%	NCEP	4	Above 13-years/994/(54.5%)	Central

31	Al-Malki et al., 2003 [50]	n/a/randomly recruited healthy females	PCC	obesity	Overweight = 189 = 31.5% Obese = 21.13%	n/a	3	16–45/600/100%	Central

32	Kalantan et al., 2001 [51]	n/a/randomly from 30 different PCC	PCC	HTN	Female = 29%	WHO-International Society of Hypertension Guidelines.	5	>35/1114/672 60% Female	Central

33	Abolfotouh et al., 2001 [52]	88%/all patients from 3 PCC in south (abha)	PCC	(1) Central obesity	(1) 29.2%	n/a	4	>+65/807/31.1%	South
				(2) HTN	(2) HTN: female = 28%				
				(3) DM	(3) DM: female = 27.1%				

34	Siddiqui et al., 2000 [53]	n/a/randomly selected	PCC	HTN	3.05%	WHO guidelines HTN	1	Mean age of females was 23.76 years/3747/55%	Central

35	Karim et al., 2000 [54]	n/a/randomly from the medical record	PCC	DM	Female 9.6%	n/a	2	<16–+75/3747/55.08%	Central

36	Al-Humaidi, 2000 [55]	n/a/3 centrals were randomly selected according to their geographical location in the city, including all patients	PCC	Obesity	Mean BMI 32.15, SD = 1.2	BMI	4	30–70/696/49.6%	South

37	Al-Qahtani et al., 2006 [56]	74.6%/all Saudi women attending PCC	PCC	(1) Mts	(1) 13.6% NCEP definition 16.1% IDF definition	NCEP- ATP III/IDF	6	18–59/2577/100%	North
				(2) Abdominal obesity	(2) 44.1% NCEP definition 67.9% by IDF				

38	Saeed et al., 2011 [57]	n/a/multistage stratified cluster random sampling technique	National all region	HTN	Female = 23.9%	WHO STEPwise approach to Surveillance of (NCD)	7	15–64/4758, 51%	All region

39	Soofi and Youssef, 2015 [14]	Included also in the study were attendees in the 2010 Cultural Festival in Riyadh		(1) Smoking	(1) 12%	Framingham Risk Score	6	20–>60/4932/55%	Central
				(2) Dyslipidimia	(2) 18%				
				(3) HTN	(3) 32%				
				(4) Obesity	(4) 27%				
				(5) DM	(5) 28%				
				(6) Physical inactivity	(6) 96%				

40	Memish et al., 2014 [58]	89.4%/Saudi Health Information Survey (SHIS) randomly selected from a national sampling	National, all region	Obesity	Overweight = 28% Obese = 33.5%	WHO, BMI	8	15–65/10,293/53.26%	All region

41	El Bcheraoui et al., 2014 [59]	89.4%/Saudi Health Information Survey (SHIS), randomly selected from a national sampling	National, all region	HTN	HTN female = 13%	National Health and Nutrition Examination Survey (NHANES) for determining blood pressure levels	9	15–65/10,293/53.26%	All region

42	Basulaiman et al., 2014 [60]	89.4%/Saudi Health Information Survey (SHIS), randomly selected from a national sampling	National, all region	High cholesterol	19.6% borderline 8.5% high	n/a	8	15 years or older/10,735/49.36%	All region

43	Aljohani, 2014 [61]	92.6%/multistage stratified cluster random sampling technique	National, all region	Mts	Female 565/2242 = 25.5%	The WHO STEPwise, IDF	8	15–64/4, 406	All region

44	Al-Daghri et al., 2014 [62]	n/a/participants were part of the Biomarkers Screening Program Database (RIYADH Cohort) patients PHCC which was taken as a cluster	All Riyadh region (RIYADH Cohort)	(1) HTN	(1) HTN = 26%	NCEP-ATP III	7	18–70/9,164/51.8% females	Central
				(2) Mts	(2) Mts = 40.3%				

45	Al-Baghli et al., 2010 [63]	93%/community-based screening campaign eastern	All eastern region screening campaign	DM	Female = 18.6%	JNC-VII	7	30–+70/197681/49%	East

46	Al-Kaabba et al., 2012 [64]	94.4%/multistage stratified cluster random sampling technique was used to recruit the study subjects	All region	High cholesterol	Female = 19.9%	WHO's STEPwise of Non-Communicable Diseases (NCD)		≥15 years/4490/51% were females	All region

47	Al-Daghri et al., 2011 [65]	n/a/patients were recruited randomly from their homes using the cluster	All Riyadh region (RIYADH Cohort)	Obesity	Female = 36.5%	(WHO) proposed cut-offs or DMT2 and Seventh Joint National Committee	6	7–80/9,149/(41.4%) 3,792	Central

48	Albedah et al., 2011 [66]	n/a/patients were recruited randomly from their homes using the cluster	All region	Smoking	2.9%	Standard international questionnaire developed by the BMRB	5	15–≥50/7003/49%	All region

49	Al-Turki et al., 2010 [67]	93%/community-based screening campaign eastern	All eastern region screening campaign	Smoking	Female 5.0%	n/a	5	30 years and above/197,681/49%	East

50	Al-Daghri et al., 2010 [68]	n/a/participants were part of the Biomarkers Screening Program Database (RIYADH Cohort) patients PHCC which was taken as a cluster	All Riyadh region (RIYADH Cohort)	Mts	Female 34.1%	NCEP ATP III	6	18–55/2850/53.2% 1515 female	Central

51	Al-Baghli et al., 2009 [69]	93%/community-based screening campaign eastern	All eastern region screening campaign	HTN	Female = 15.6%	JNC7	6	>30/197,681/49%	East

52	Al-Turki et al., 2008 [70]	93%/community-based screening campaign eastern	All eastern region screening campaign	HTN	Female = 18.1%	JNC7	6	>30/197,681/49%	East

53	Al-Nozha et al., 2008 [71]	n/a/subjects were selected using a 2-stage stratified cluster sampling procedure, urban and rural being the strata.	National, all region	High cholesterol	Female TC > 5.2 mmol = 53.2% Female >TG = 33.7%	NCEP, ATP III	8	30–70/16819/52.3%	All region

54	Al-Baghli et al., 2008 [72]	93%/community-based screening campaign eastern	All eastern region screening campaign	Obesity	Overweight female 29.7% Obese female 51.8%	NIH study identifies ideal body mass index	6	>30/195,874/49%	East

55	Al-Othaimeen et al., 2007 [73]	n/a/random house hold selection	National, all region	Obesity	Overweight female 28.4% Obese female 23.6%	World Health Organization (WHO) BMI	5	≥18/17,892, 51.5% female	All region

56	Al-Nozha et al., 2007 [74]	98%/subjects were selected using a 2-stage stratified cluster sampling procedure, urban and rural being the strata	National, all region	Physical inactive	98.1%	Metabolic equivalent: MET	6	30–70/17395/52.3%	All region

57	Al-Nozha et al., 2007 [75]	n/a/subjects were selected using a 2-stage stratified cluster sampling procedure, urban and rural being the strata	National, all region	HTN	Female 2148/9006 = 23.9%	JNC7	6	30–70/17,230/52.3%	All region

58	Al-Nozha et al., 2005 [76]	n/a/subjects were selected using a 2-stage stratified cluster sampling procedure, urban and rural being the strata	National, all region	Obesity	31.8% overweight 44.0% obese	WHO BMI	6	30–70/17,232/52.3%	All region

59	Al-Nozha et al., 2005 [77]	n/a/subjects were selected using a 2-stage stratified cluster sampling procedure, urban and rural being the strata	National, all region	Mts	Female: 22%	ATP III	6	30–70/17,232/52%	All region

60	Al-Nozha et al., 2004 [78]	98.2%/2 stage stratified cluster sampling procedures, urban and rural being the strata	National, all region	DM	Female: 21.5%	ADA, Hg A1C > 7 mmol	6	30–70/17,232/52%	All region

61	Moradi-Lakeh et al., 2015 [79]	95.88%/Saudi Health Interview Survey was a cross-sectional national multistage survey of individuals aged ≥15, and households were randomly selected from each block	National, all region	Smoking	Female: 1.1%	They asked for current use and current daily smoking of tobacco products	7	49.4%	All region

*Q* score = quality score, n/a = not available, Mts = metabolic syndrome, DM = diabetes mellitus, HTN = hypertension, JNC7 = Joint National Committee, WHO = World Health Organization, NCEP = National Cholesterol Education Program, High TCh = high total cholesterol.
