# Supplementary material for: Women in Saudi Arabia and the Prevalence of Cardiovascular Risk Factors: A Systematic Review
Source: J Environ Public Health. 2016 Sep 29;2016:7479357. doi: 10.1155/2016/7479357 (PMC5061969; doi:10.1155/2016/7479357)
Supplement: Supplementary file 1 — Newcastle–Ottawa scale (NOS) is a tool used for assessing the quality of non-randomized studies included in a systematic review and/or meta-analyses. NOS awards a maximum of nine stars to each study: four stars for the adequate selection of cohort studies, two stars for comparability of cohort studies on the basis of the design and analysis, and three stars for the assessing the outcome. [file 7479357.f1.pdf]

Supplement report

Table 2**: Quality assessment

Quality Assessments

Number	study	Selection total = total 4 points												Comparability= total 2 points		Outcome= total 3 points						Total=9
		1.Representativeness of the sample				2.Sample size		3.Non-respondents			4.Ascertainment of the exposure			1.Confounding factors		1.Assessment				2.Statistical		
		A*	B*	C	D	A*	B	A*	B	C	A*	B*	C	A*	B*	A*	B*	C*	D	A*	B	
1	Mahfouz, Alsanosy, Gaffar, & Makeen, 2014	*				*						*		*				*		*		6
2	Khalaf, Westergren, Berggren, Ekblom, & Al-Hazzaa, 2014		*			*						*		*	*			*		*		7
3	Ibrahim et al., 2014		*			*					*			*	*			*		*		7
4	AlSwuailem, AlShehri, & Al-Sadhan, 2014		*				*						*	*	*			*		*		5
6	Koura, Al-Dabal, Rasheed, Al-Sowielem, & Makki, 2012		*			*				*	*				*			*		*		6
7	Azhar & Alsayed, 2012		*				*			*	*				*			*			*	3
8	Allam, Taha, Al-Nozha, & Sultan, 2012		*				*			*	*				*			*		*		5
9	Abolfotouh, Al-Alwan, & Al-Rowaily, 2012		*				*			*			*		*				*	*		3
10	Wali, 2011		*				*			*	*				*			*		*		5
12	Mandil et al., 2011		*				*		*		*				*			*		*		5
13	Al Qauhiz, 2010		*			*				*			*		*			*		*		5
14	Subhan, Al-Khlaiwi, & Ghandourah, 2009		*				*			*			*		*			*		*		4
15	Y. A. Al-Turki & Al-Rowais, 2008			*			*						*					*			*	1
16	Merdad, Al-Zahrani, & Farsi, 2007		*			*				*	*				*			*			*	5
17	Hasim, 2000			*			*			*			*		*			*				2
18	Amin, Al Sultan, Mostafa, Darwish, & Al-Naboli, 2014	*					*				*			*	*		*	*		*		7
19	Rehmani et al., 2013	*				*						*		*	*		*				*	6
20	Siddiqui & Ogbeide, 2001																					
21	Abalkhail, Shawky, Ghabrah, & Milaat, 2000		*			*							*		*			*		*		5
22	AlQuaiz et al., 2015				*		*			*	*			*	*			*		*		5
23	Elkhalifa, Kinsara, & Almadani, 2011			*			*			*			*		*			*		*		3

24	Al-Daghri et al., 2013		*				*			*		*			*				*	*		4
25	Habib, 2013				*		*			*			*					*				1
26	Amin, Al-Hammam, et al., 2014	*									*			*	*			*		*		7
27	Ahmed, Ginawi, Elasbali, Ashankyty, & Al-Hazimi, 2014	*				*						*			*	*	*			*		7
28	Safar A Al-Saleem, 2013		*			*							*	*	*		*					5
29	Al-Zahrani, 2011				*		*			*			*		*			*		*		3
30	Alqurashi, Aljabri, & Bokhari, 2011				*		*			*			*		*			*		*		3
31	Ogbeide, Karim, Al-Khalifa, & Siddique, 2004				*		*			*			*		*			*				2
32	Al-Malki, Al-Jaser, & Warsy, 2003				*		*			*		*			*		*				*	3
33	Kalantan, Mohamed, Al-Taweel, & Abdul Ghani, 2001		*			*				*			*		*		*			*		5
34	Abolfotouh, Daffallah, Khan, Khattab, & Abdulmoneim, 2001		*				*			*			*		*		*			*		4
35	Saima Siddiqui, 2000				*		*			*			*				*					1
36	Karim, Ogbeide, Siddiqui, & Al-Khalifa, 2000		*				*			*			*				*					2
37	Al-Humaidi, 2000				*		*			*	*			*			*			*		4
38	Al-Qahtani, Imtiaz, Saad, & Hussein, 2006		*			*				*	*				*		*			*		6
39	Saeed et al., 2011	*				*				*	*			*	*		*			*		7
40	Memish et al., 2014	*				*		*				*		*	*	*				*		8
41	El Bcheraoui et al., 2014	*				*		*				*		*	*		*	*		*		9
42	Basulaiman et al., 2014	*				*		*				*		*	*	*				*		8
43	Aljohani, 2014	*				*					*			*	*	*	*			*		8
44	Al-Daghri et al., 2014	*				*					*			*	*		*			*		7
45	Al-Baghli et al., 2010		*			*		*				*			*			*		*		7
46	Al-Daghri et al., 2011		*			*				*		*			*		*			*		6
47	Albedah, Khalil, Khalil, & Elolemy, 2011		*			*				*			*		*			*		*		5
48	K. A. Al-Turki et al., 2010		*			*				*		*			*			*		*		5
49	Al-Daghri et al., 2010		*			*				*		*			*			*		*		6
50	Al-Baghli et al., 2009		*			*				*		*			*			*		*		6

51	K. A. Al-Turki, Al-Baghli, Al-Ghamdi, & El-Zubaier, 2008		*			*				*		*			*			*		*		6
52	Al-Nozha et al., 2008	*				*		*				*		*	*		*			*		8
53	Al-Baghli et al., 2008		*			*				*		*			*		*			*		6
54	Al-Othaimeen, Al-Nozha, & Osman, 2007		*				*					*			*			*		*		5
55	Al-Nozha, Al-Hazzaa, et al., 2007	*				*				*		*			*			*		*		6
56	Al-Nozha, Abdullah, et al., 2007	*				*				*		*			*			*		*		6
57	M. M. Al-Nozha et al., 2005	*				*				*		*			*			*		*		6
58	M. Al-Nozha et al., 2005	*				*				*		*			*		*			*		6
59	Al-Nozha et al., 2004	*				*				*		*			*			*		*		6
60	(Soofi & Youssef, 2015)		*			*						*		*				*		*		6
61	(Moradi-Lakeh et al., 2015)	*	*			*					*				*			*		*		7
